# Internal Habitat Quality Determines the Effects of Fragmentation on Austral Forest Climbing and Epiphytic Angiosperms

**DOI:** 10.1371/journal.pone.0048743

**Published:** 2012-10-31

**Authors:** Ainhoa Magrach, Asier R. Larrinaga, Luis Santamaría

**Affiliations:** Laboratory of Spatial Ecology. Mediterranean Institute for Advanced Studies, Esporles, Mallorca, Balearic Islands, Spain; University of Western Australia, Australia

## Abstract

Habitat fragmentation has become one of the major threats to biodiversity worldwide, particularly in the case of forests, which have suffered enormous losses during the past decades. We analyzed how changes in patch configuration and habitat quality derived from the fragmentation of austral temperate rainforests affect the distribution of six species of forest-dwelling climbing and epiphytic angiosperms. Epiphyte and vine abundance is primarily affected by the internal characteristics of patches (such as tree size, the presence of logging gaps or the proximity to patch edges) rather than patch and landscape features (such as patch size, shape or connectivity). These responses were intimately related to species-specific characteristics such as drought- or shade-tolerance. Our study therefore suggests that plant responses to fragmentation are contingent on both the species' ecology and the specific pathways through which the study area is being fragmented, (i.e. extensive logging that shaped the boundaries of current forest patches plus recent, unregulated logging that creates gaps within patches). Management practices in fragmented landscapes should therefore consider habitat quality within patches together with other spatial attributes at landscape or patch scales.

## Introduction

Habitat fragmentation has been described as one of the major drivers of biodiversity loss worldwide [1]–[6], particularly in the case of forest ecosystems, which are decreasing globally at an alarming rate [7]–[9]. Forest fragmentation may affect forest-dwelling organisms through several (though not necessarily independent) pathways, including the effects of decreasing patch size, increased patch isolation, altered habitat conditions [10]–[14] and the alteration of plant-animal interactions (e.g. [15]–[16]). Though most research has focused to date on animal populations, several studies have shown that plant populations tend to be smaller and show decreased reproductive outputs (seed production and germinability) in fragmented than in continuous habitats [15],[17]–[19]. These population changes result in a higher extinction risk due to the combined effects of higher demographic stochasticity and increased isolation between local populations [20].

Plants persisting in forest fragments may also be affected by “edge effects”, caused by structural and microclimatic changes at the interface between the forest interior and the human-altered matrix outside it [21]–[22]. The penetration distance of these edge effects (the “edge width”) is related to the nature of both the perturbation causing forest fragmentation and the altered landscape “outside” the patches. However, it also depends on patch size and shape [23]–[25], rendering it desirable to measure all of these effects jointly.

The approaches taken by researchers to address the effect of fragmentation range from landscape-scale studies [26] to others that focus on local-habitat characteristics of remnant forests [27]. Most of these studies fail, however, to consider the multiple components of the fragmentation process and the variety of spatial scales at which these operate (but see [28]). Discriminating the effects of each of these components and their operational scales is a necessary step to obtain a causal understanding of the effects of fragmentation, because equivalent spatial patterns can be caused by totally different processes [29]–[30]. In addition, previous studies have shown that such responses may vary greatly among different organisms and species, making it essential to undertake multi-species comparisons (see [31]).

Although a major part of the fragmentation literature pays limited attention to the role of habitat quality within the fragments, this is a variable classically analyzed in the conservation literature (see for example [32], [33]). In particular, owing to their sessile habit, plants are likely to be specially sensitive to changes in (micro)habitat characteristics taking place during or after the process of fragmentation, especially when the latter is associated to both natural or anthropogenic disturbance (e.g. logging, fires, etc.).

In this study, we assess the relative effects of six different components of forest fragmentation (inter-patch connectivity, patch size, patch shape, local abundance of biotic dispersers, habitat quality and the distance to the nearest forest edge) on the distribution of forest-dwelling plants that depend on trees at some point in their life cycle for support (including vines, epiphytes and a mistletoe). Descriptors of habitat quality focused on tree size, since it provides a measure of habitat availability both in space and time (i.e. larger trees tend to be older); the abundance of logs and snags, since they provide alternative colonization substrates; and the clearing of internal areas of the forest by unregulated logging (hereafter “logging gaps”).

Climbing and epiphytic angiosperms are especially amenable for studies of habitat fragmentation, because the necessity of a specific substrate makes their survival strongly linked to the presence of host trees. In this study, we surveyed the distribution of all species of mistletoes, climbing and epiphytic angiosperms (excluding shrubs and facultative epiphytes) inhabiting our study system –the austral temperate forests of Southern Chile. The species present in this area differ largely in physiological, morphological and reproductive characteristics, including their main pollinators and seed dispersers [34]–[35]; hence, we expected them to respond differently to forest fragmentation [25]. In particular, we hypothesized that the abundance and species richness of climbing and epiphytic angiosperms (mistletoe, epiphytes and vines) will depend on landscape- and patch-level characteristics (increasing with patch size and decreasing with patch isolation), on within-patch characteristics (decreasing nearby patch edges and logging gaps, and increasing with host-tree size; e.g. [36]), and on the abundance of mutualistic species (seed dispersers).

## Results

We recorded 8 species of mistletoes, climbing and epiphytic angiosperms in the surveyed forests: *Mitraria coccinea, Sarmienta repens, Asteranthera ovata, Luzuriaga polyphylla, Luzuriaga radicans, Campsidium valdivianum, Tristerix corymbosus* and *Fascicularia bicolor* (Table S1 in Supplementary Material). However, the low prevalence found for the last two species (only 3 and 17 individuals of, respectively, *T. corymbosus* and *F. bicolor* found across the complete survey) did not allow us to analyze their distribution.

**Figure 1:**
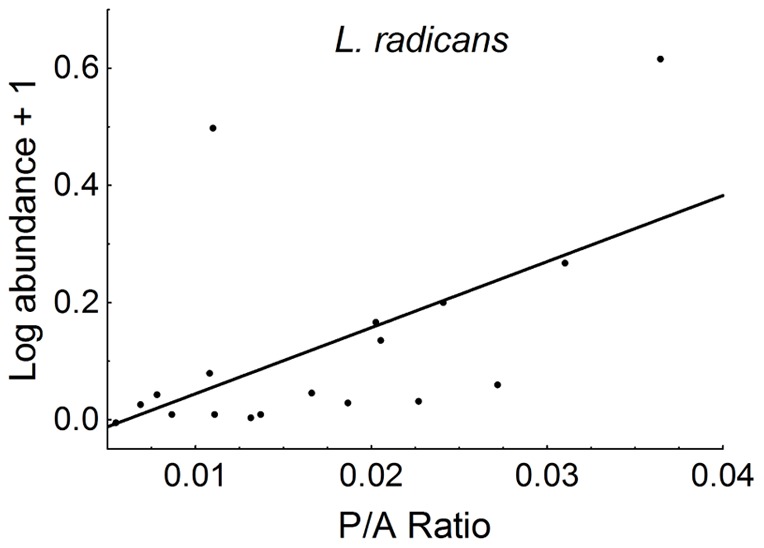
Partial residual plots showing the effects of patch shape on the patch-level abundance of *Luzuriaga radicans*. Filled circles represent the sum of average predicted and residual values per patch.

The results of the analyses show that, in the fragmented forests of Chiloé Island, the abundance of climbing and epiphytic angiosperms is influenced by transect-level (i.e. within-patch) rather than patch- and landscape-level characteristics (Table 1). The only patch-level variable present in the reduced models was the perimeter/area ratio (P/A RATIO), and it only affected significantly one of the six species studied (*L. radicans*, whose abundance increased in irregularly-shaped patches; Fig. 1).

**Table 1:** Results of General Lineal Mixed Models describing the effect of patch- and transect-level environmental variables on the abundance of six epiphytes and vines present in 18 forest fragments at Chiloé Island (S Chile).

	Patch-level	Transect-level						
	P/A RATIO	D-EDGE	D-EDGE^2^	LogDBH	LogDBH^2^	GAP	TREES	LogDBH* TREES
*L. radicans* ^∫^	11.3±4.7*			(6.9±5.2)^e−2^ ^NS^				
*L. polyphylla* ^§^	0.40±5.47 ^NS^	(9.7±1.7)^e−3^ ***	(−8±2)^e−5^ ***	−0.91±0.33 **	0.56±0.14 ***			
*M. coccinea* ^§^	5.56±3.95 ^NS^			0.20±0.09 *			(−3.6±1−1)^e−2^ **	(7.4±1.4)^e−2^ ***
*S. repens* ^†^				3.31±0.59 ***				
*C. valdivianum* ^†^						1.20±0.31 **		
*A. ovata* ^†^	5.46±14.85 ^NS^					−1.27±0.33 **		
DBH	0.67±1.80 ^NS^	(4.5±1.8)^e−4^ *						

Results of a model describing the effect of all other independent variables on tree DBH, the best predictor of epiphyte abundance, are also provided. Figures represent parameter estimates (estimate ± standard error) and significance levels for the variables retained in the best (reduced) model. Variables excluded from the reduced models in all cases (i.e. for all species) are not shown in the table. ∫ Epiphyte abundance  =  number of ramets per tree (N = 2467 at transect level).§Epiphyte abundance  =  number of ramets per 10-m transect. † Epiphyte presence  =  proportion of trees occupied per 10-m of transect (30 m for *A. ovata*; N = 680 and N = 197 at transect level, respectively). Patch level: N = 18. NS P>0.10, * P<0.05, ** P<0.01, *** P<0.001.

**Figure 2:**
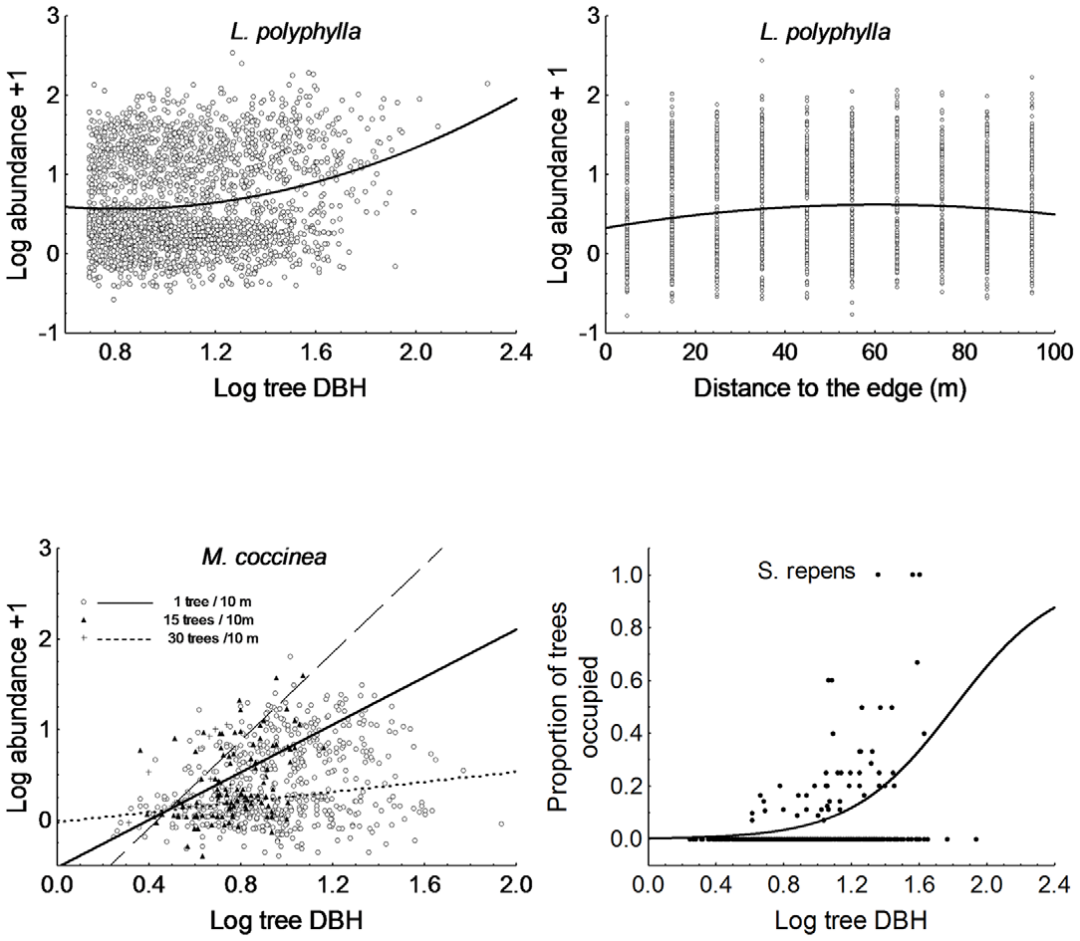
Partial residual plots showing the effects of within-patch habitat characteristics on the abundance of different species. (a) and (b) *Luzuriaga polyphylla*, (c) *Mitraria coccinea*, and (d) *Sarmienta repens*. Graph (c) shows the relationship between epiphyte abundance and Log(tree DBH) for several values of tree density.

**Figure 3:**
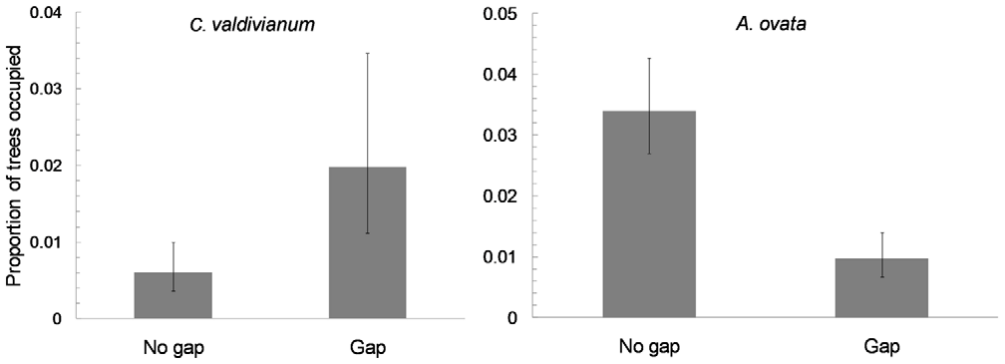
Effect of gaps created by unregulated logging within forest patches on the abundance of two species. (a) *Campsidium valdivianum* and (b) *Asteranthera ovata*.

In contrast, transect-level variables were selected in all reduced models (Table 1). The most important were tree size (DBH), with significant, positive effects on the abundance of three species (*L. polyphylla*, *M. coccinea* and *S. repens*; Fig. 2); and logging gaps (GAP), with significant effects on two species (*C. valdivianum* and *A. ovata*) that were, respectively, more and less abundant in gaps (Fig. 3). The effect of DBH tended to saturate and decrease at large DBHs for *L. polyphylla* (significant quadratic term; Table 1) and interacted with the number of trees for *M. coccinea* – leveling off as tree density decreased (Table 1, Fig. 2). Finally, the distance to the nearest edge (D-EDGE) showed a quadratic effect on *L. polyphylla* – whose abundance increased from the edge to approx. 40–60 m inside the patch, but decreased again when progressing further towards the patch center (Table 1, Fig. 2).

We also found a significant, negative effect of the distance to the nearest edge (D-EDGE) on tree DBH, with larger trees being found farther away from the patch edge (Table 1). The density of the main pollinator and the avian dispersers did not affect the distribution of any of the species surveyed.

## Discussion

Our results show that local-scale (within-patch) rather than patch- and landscape-scale characteristics determine the abundance of climbing and epiphytic angiosperms in fragmented, austral temperate forests. Contrary to our expectations, the only relationship found between patch-scale variables and plant abundance involved a single species (*L. radicans*) and a single variable (perimeter/area ratio). Moreover, because this variable is strongly, negatively related to both patch size (in our dataset: Spearmańs rho = 0.96) and edge/core area ratio (e.g. [37]), its positive relationship with *L. radicans*' abundance may well reflect the compounded, positive effects of decreased patch size and an increased proportion of edge area relative to core areas (characterized e.g. by higher light availability) rather than the sole effect of patch shape.

In contrast, several variables describing within-patch habitat quality had considerable effects on the six study species. The most important was host-tree size, as described by its diameter at breast height (DBH), which correlated positively with the abundance of three out of six epiphytes. This effect probably reflects the longer colonization period of larger (therefore older) trees, though reduced competition (i.e. larger colonization surface) and enhanced microhabitat conditions (e.g. availability of sites closer to the canopy or aging effects on the trees' bark) could also play a role. Similar previous studies carried out in the same area did not find such an effect [34], however these authors recorded data on epiphytes and vines from plots located more than 200 m from the nearest edge, in contrast to our study featuring edge-to-interior transects. It is precisely this gradient that makes the difference. Edge effects influenced the abundance of three species through either direct effects (one species, *L. polyphylla*, whose abundance peaked at 40–60 m from the edge, although large scatter in our data suggests this result be interpreted with caution)) or indirect ones (mediated by tree size on *L. polyphylla* and *M.coccinea*). The decrease in tree DBH towards the edge indicates that fragments are undergoing an “edge sealing” process; whereby, enhanced tree recruitment near the edges [22] gradually shortens the extension and magnitude of edge penetration in forests and reduces its effects on epiphytes.

Second in importance was a measure of habitat degradation, the presence of gaps caused by unregulated logging inside forest patches, which had positive effects on the vine *C. valdivianum* and negative effects on the epiphyte *A. ovata*. These effects show a reasonable match with the species' ecology: *A. ovata* is a low-height, shade-tolerant, drought-intolerant species [38] that probably suffers under or nearby canopy openings, while *C. valdivianum* is a climbing liana that probably benefits from the increased sunlight created by logging gaps (as in [39]).

The effects of forest fragmentation at the within-patch scale have been rarely reported, probably owing to several reasons. Firstly, most studies and models have focused on animals that show strong effects at the landscape and patch-scales (e.g. [12], [40]). Plants, which have received much less attention (although see review in [19]), show a number of characteristics (greater longevity, sporadic recruitment, frequent asexual reproduction, ability to survive persistent unfavorable conditions; [19]) that make their individual and population responses different from those of animals. For example, the greater longevity of plants is likely to result in delayed population responses, facilitating the existence of “extinction debt” effects (i.e. when the extinctions occur generations after fragmentation took place; [41]).

Secondly, studies reporting significant effects of patch area and connectivity on plants largely focus on species diversity and/or community structure [28], and are therefore highly sensitive to the effect of rare species present only in large connected patches [28]. Our study deals with the abundance of relatively common climbing and epiphytic angiosperm species, which may show slower responses than rare species and therefore be more prone to extinction-debt effects, suggesting that our results must be considered with caution. At any rate, our study species showed a much stronger sensitivity towards local habitat conditions, which suggests that this is the pathway more likely to affect their persistence in the near future.

In addition to the direct effects of patch-scale factors, we evaluated the existence of indirect effects caused by the dependence of our focus species on a narrow set of pollinators or dispersers with contrasting responses to forest fragmentation. None of the patterns observed confirmed this hypothesis. Because we have observed strong effects of patch shape, patch connectivity and distance to the edge on the abundances of key pollinators (hummingbird) and dispersers (the marsupial, *D. glirioides*, [42], and two frugivorous birds, *Elaenia albiceps* and *Turdus falcklandii*; [31] and personal observations), this result suggests that abundance of these climbing and epiphytic angiosperms is relatively insensitive to the abundances of animal vectors – either because they do not influence reproductive rates, or because effects on reproductive rates do not translate in changes in the abundances of climbing and epiphytic angiosperms over the short term. The second is a likely possibility, given that the fragmentation of Chiloé's forests is relatively recent [43] and population changes may have lacked time to build up. Further research will explore these possibilities, by analyzing the effects of changes in pollination and disperser abundance on epiphyte reproduction.

Overall, our study suggests that the responses of plants to fragmentation are contingent on both the species' ecology and the specific pathways through which the study area is being fragmented. In our case, forest recruitment at patch edges overlapped with the within-patch impact of unregulated logging. The result was a mosaic of indirect responses to external edges (mediated by host-tree size) and direct responses to internal gaps, which interacted with the specific ecology of the different climbing and epiphytic angiosperm species to generate a variety of responses, broadly characterized by the predominance of habitat-quality factors over patch configuration (as also shown in previous studies, see [36]).

Given the high risk facing the studied forests, which suffer a very high rate of unregulated logging, we believe our study (though preliminary) has considerable applied importance. Our results highlight the importance of maintaining not only landscape connectivity or patch size, but also internal patch quality. The informal nature of current logging practices in response to the high demand for fuel wood [44] that predominate in Chiloé results in the “internal” degradation of apparently intact patches. Our results suggest that addressing this issue is of key importance to safeguard forest biodiversity in this area (as shown in [45]). The take-home message is that management practices in altered landscapes should not concentrate exclusively on landscape or patch attributes (without questioning their demonstrated importance), but should seriously consider habitat quality within patches (as suggested by [32]) – particularly when the conservation of sessile organisms is at stake.

## Materials and Methods

### Study site and species

**Figure 4:**
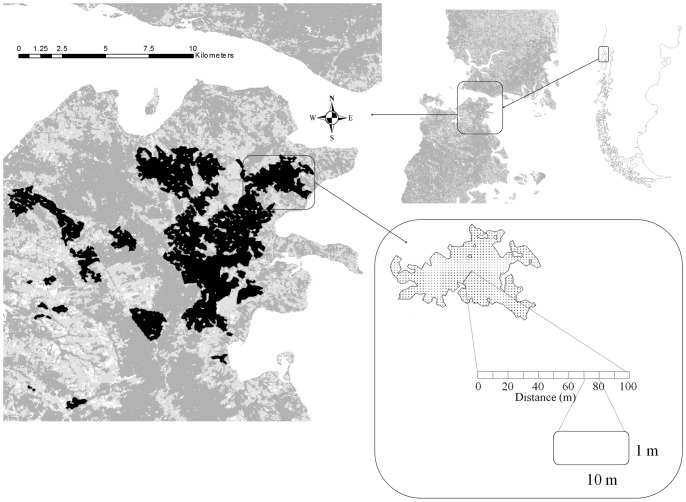
Study area and sampling design. Shaded areas indicate forest fragments, black areas indicate the 18 fragments included in the survey. Inset (lower-right corner) shows one of these fragments with a schematic representation of transects and segments used for the survey.

The study site was located at Isla Grande de Chiloé, southern Chile (42°00′ S, 73°35′ W, Fig. 4), a 9,181 km^2^ island originally covered by a mixture of Valdivian and North Patagonian temperate rainforests, currently considered among the world´s 200 most endangered ecoregions [46]. Old-growth and secondary forests are dominated by broad-leaved evergreen species with an abundant understory of bamboo (*Chusquea* sp.), numerous logs and snags, and shrub-dominated degradation stages in gaps and open areas (see [47]). Chiloé's forests are also characterized by the high abundance, diversity and endemicity of vines and epiphytes (e.g. they host over 50 species of epiphytic bryophytes, ferns and angiosperms, many of which are endemic, including four monotypic genera; [38], [48]). Angiosperm climbing and epiphytic plants are key resources for local pollinators: they provide 67% of the flowers visited by the main, and almost exclusive, local bird pollinator (the green-backed firecrown hummingbird *Sephanoides sephaniodes*, Trochilidae), and their flowers show the highest nectar volume, sugar concentration and energetic content of all the local bird-syndrome flowers [49]. Climbing and epiphytic angiosperms are also key resources for local frugivores (72% of their species bear fleshy fruits, and represent 27% of Chiloé's plant species with bird-dispersed syndrome), a characteristic that differentiates these forests from other temperate and neotropical areas, where most vines and epiphytes are wind-dispersed [50].

Over the last two centuries, human activities at Chiloé Island have converted two-thirds of its original forest into crops and pastures – a process that has accelerated greatly during the last three decades owing to agricultural expansion and the increasing demand of fuel wood [43], [44]. Associated landscape-scale modifications include an increasing number of forest patches with decreasing sizes, more irregular shapes and longer edges [43].

Our work focused on the community of angiosperm climbing and epiphytic plants, including holoepiphytes (*sensu* [51]), secondary hemi-epiphytes and the vine *Campsidium valdivianum*, but excluding facultative epiphytes (such as *Griselinia racemosa, Pernettya insana* and *Philesia magellanica*) and the hemi-epiphytic shrub *Pseudopanax laetevirens*. We recorded eight species of climbing and epiphytic angiosperms in the surveyed forests: *Mitraria coccinea*, *Sarmienta repens*, *Asteranthera ovata*, *Luzuriaga polyphylla*, *Luzuriaga radicans*, *Campsidium valdivianum*, *Tristerix corymbosus* and *Fascicularia bicolor* (Table S1 in Supplementary Material). *Mitraria coccinea* (Cav.), *Sarmienta repens* (Ruiz & Pav.) and *Asteranthera ovata* (Cav.) Hanst. (Gesneriaceae) produce red tubular flowers and greenish fleshy-fruits with many small seeds. *M. coccinea* and *A. ovata* grow as hemi-epiphytes, while *S. repens* grows as an holoepiphyte ([35]; Table S1). *Luzuriaga polyphylla* (Hook.) and *Luzuriaga radicans* (Ruiz & Pav.) (Philesiaceae) bear bell-shaped, insect-pollinated white flowers [52], produce fleshy fruits and grow as hemi-epiphytes. *Campsidium valdivianum* (Phil.) (Bignoniaceae) is a vine that belongs to a monotypic genus endemic to South-American temperate forests, bears pink tubular flowers with deep corollas, and produces dehiscent, dry fruits with wind-dispersed seeds. *Tristerix corymbosus* (Kuijt) (Loranthaceae), is a hemi-parasitic mistletoe that presents inflorescences of conspicuous red, tubular flowers which turn into single-seeded green berries [53] consumed solely by the marsupial *Dromiciops gliroides*, whose gut treatment is required for successful seed germination and seedling establishment [54]. *Fascicularia bicolor* (Ruiz & Pav.) Mez. (Bromeliaceae) shows a green foliage that turns brilliant red before and during blooming, and large tight clusters of blue flowers that turn into berries with many ovoid-shaped seeds [55].

### Epiphyte surveys

We surveyed the abundance of epiphytes and vines at 18 forest fragments chosen to differ largely in size, shape and connectivity (Fig. 4, see Table S2). The fragments selected in the study have been isolated from each other for at least 23 years (comparison between two Landsat TM images, one contemporaneous to our surveys, taken on February 18, 2008 and one 23 years older, taken on January 25 1985). Although we lack data on species longevity, the long history of patch isolation suggests that the populations studied are not a persistent subset of pre-fragmentation populations, but viable populations able to maintain themselves through the successful reproduction and establishment of new individuals.

For this purpose, we used four linear transects (100 m long by 1 m wide, or as long as patch geometry and size allowed for some of the patches studied) departing at each fragment's edge and progressing towards its center. Whenever patch size and geometry allowed (i.e. for all but six of the patches studied), minimum distance between transects was >100 m.

Throughout the longitudinal transects we measured some microhabitat characteristics that could be affecting the distribution of the climbing and epiphytic angiosperms surveyed. Because living trees represent their main substrate, we included tree size (DBH, a measure of both substrate quality and, owing to its correlation with tree age, time for colonization, [38 and Piazzon et al unpublished data]). For every tree, we recorded the abundance of climbing and epiphytic angiosperms growing on its stem and branches. For *L. polyphylla*, *L. radicans* and *M. coccinea*, abundance was measured as the number of ramets per host tree. For *S. repens*, *A. ovata* and *C. valdivianum*, plant architecture did not allow for a reliable identification of individual ramets; hence, we only recorded their presence or absence per host tree.

Along each transect, distance to edge (D-EDGE) was recorded at 10-m intervals. Within each interval, we also recorded the presence of native bamboo thickets (*Chusquea* sp.; QUILA), a key determinant of the characteristics of the understory, that conditions the potential for establishment of vines and hemi-epiphytes (which recruit in the forest floor) as well as the abundance of key seed dispersers (the marsupial *Dromiciops gliroides*). For every 10-m interval, we also recorded the presence of logging gaps (caused by unregulated logging within the forest patches; GAPS) because they have a known effect on several key variables, including light availability, wind exposure and desiccation stress, as well as the number of logs (LOGS) and snags (SNAGS) because they represent alternative substrates and provide a useful surrogate of forest disturbance.

### Patch metrics

Patch metrics were obtained from a Landsat TM image (February 18, 2008) analyzed using an iso-data, non-supervised algorithm based on a 20-class categorization (Idrisi 15.0, Andes Edition XX). These classes were then grouped into two categories, representing forested and non-forested areas, adjusted to match our field observations. The resulting layer of forested areas was then analyzed using FragStatsBatch for Arcgis 9 [56]–[57] and V-LATE 1.1 for Arcgis 9 [58], to produce a series of descriptive measures for every forest fragment: patch size, two measures of patch shape (CIRCLE and P/A RATIO) and two measures of patch isolation (PROX and DIST). CIRCLE is defined as 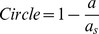, where *a* is the area of the focal patch and *a_s_* is the area of the smallest circumscribing circle around the patch; it takes values of 0 for circular patches and tends to 1 for elongated, linear patches one-cell wide [56]. P/A RATIO is the ratio between patch perimeter and area; it increases as patch shape becomes more irregular. DIST is the Euclidean distance to the nearest neighbor. Proximity index, PROX, is defined as 
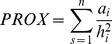
, where *a_i_* is the area of every patch *i* falling within specified neighborhood “buffers” of the focal patch (see below) and *h_i_^2^* is the edge-to-edge distance between each patch *i* and the focal patch [56]. After preliminary calculations for different buffer sizes around the focal patches (ranging from 100 to 2000 m), we selected the buffer size at which the value of the proximity index saturated (500 m).

### Pollinator and disperser densities

We surveyed the same forest fragments to estimate the densities of the main avian pollinator (the hummingbird *Sephanoides sephaniodes*, pollinates 4 of the 6 species surveyed) and dispersers (the sum of *Turdus falklandii* and *Elaenia albiceps* densities, disperse two of the 6 species sampled).

At each forest patch, we located 8 census points: four (hereafter referred to as “patch-centre”) points were situated at 100 m from the nearest edge (or as far as patch geometry and size allowed for some of the fragments studied) and separated by a minimum distance of 100 m from each other (i.e. for all but six of the patches studied); the other four points were located between each of the previous points and the forest edge, and randomly assigned to four distances to such edge (one each): 0, 25, 50 and 75 m.

At each census point, we allowed five minutes for birds to settle between our arrival and the start of the bird count, and then recorded for eight minutes every cue (visual or songburst) belonging to the afore-mentioned bird species, together with an estimate of the distance between the registered cue and the observeŕs point [59]. To ensure the reliability of distance estimates, the observer performing the survey undertook a period of training in one of the fragments surveyed, using a laser-based rangefinder (Nikon 550 AS) to compare the estimated distances with reference measures. To account for possible differences in the detection probability, we also measured the number of trees present in a 10-m-strip around each census point and introduced it as a covariate in the analyses (see below).

Finally, with the aim of obtaining reference values of cue-rates for the three bird species, we also measured the number of songbursts produced per time unit in a subsample of focal individuals whose position allowed for clear, simultaneous visual observations during at least five minutes.

### Data Analyses

We estimated bird densities using the MCDS module from program Distance 5.0 ([60], one project per species). We fitted a global model with surveyed points as strata and the mean number of trees per 10 m transect-segment as a covariate. We also included species cue-rate to obtain bird densities from the number of cues.

We obtained global model fits for the two key functions available in MCDS engine (half-normal and hazard-rate with different adjustment terms), selected the best-performing model based on their AIC value and used such model to carry out bootstrap calculations of global-level variance (based on 1000 resamples) and to estimate point-level bird densities [61].

Climbing and epiphytic angiosperm abundances (or presences) were fitted, separately for each species, to Generalized Linear Mixed Models (PROC GLIMMIX in SAS 9.1; SAS Institute, Gary, NC, 2002–2003). Abundances of *L. polyphylla*, *L. radicans* and *M. coccinea* were log-transformed and fitted using normal error distributions and identity links. Presences of *S. repens*, *A. ovata* and *C. valdivianum* were grouped for each 10-m interval (30-m intervals for *A. ovata*) to ensure model convergence, and fitted using binomial distributions and logit links. Initial models included independent variables defined at landscape (PROX and DIST), patch- (AREA, P/A RATIO, CIRCLE, POLLINATOR and DISPERSER) and transect-level (D-EDGE, logDBH, GAP, QUILA, LOGS and SNAGS), plus “patch” and “transect” random factors to define the levels of replication of the corresponding variables. Initial models were reduced by fitting the complete family of nested sub-models (full-model plus all the potential subsets of independent variables) and selecting the one with the lowest Corrected Akaike Information Criterion (AICc, hereafter). Previous to fitting each model, we identified all groups of strongly-correlated independent variables (r>0.65) and removed them from the full model; instead of selecting a single variable to represent each of these groups (based e.g. on univariate fits), we fitted separate full models for each variable of the group (and their sub-models) and selected the best-performing model based on the AICc.

Dependent-variable values and raw residuals (of the reduced model) were analyzed to evaluate the existence of spatial autocorrelation. Morańs I was calculated at both the transect- and the patch-level using SAM v3.1 [62], with distance units respectively ranging from 0 to 100 m and from 1 to 19 km. We found no significant autocorrelation in the residuals of any of the reduced models; hence, we can assume that the significant spatial autocorrelations found in the raw dependent variables (species' abundance or presence) was adequately explained by the environmental variables introduced in the models (see Table S3 in Supplementary Material).

## Supporting Information

Table S1 **Summary characteristics of epiphytes and vines found in the surveyed forests. Data collected by the authors or reviewed from** [**63**] **and** [**38**] **.**
(DOC)

Table S2 **Values for some of the variables measured at the patch level for each of the 18 patches sampled.**
(DOC)

Table S3 **Spatial autocorrelation analyses.** Correlograms showing Morańs I statistic as a function of the distance between trees inside patches (distances ranging from 0 to 100 m) and between total patches (distances ranging from 0 to 20 km). a) *L. radicans* b) *L. polyphylla* c) *M. coccinea* d) *S. repens* e) *C. valdivianum* f) *A. ovata*.
(DOC)
