# Supplementary material for: Internal Habitat Quality Determines the Effects of Fragmentation on Austral Forest Climbing and Epiphytic Angiosperms
Source: PLoS One. 2012 Oct 31;7(10):e48743. doi: 10.1371/journal.pone.0048743 (PMC3485344; doi:10.1371/journal.pone.0048743)
Supplement: Table S1 — Summary characteristics of epiphytes and vines found in the surveyed forests. Data collected by the authors or reviewed from [63] and [38] . (DOC) [file pone.0048743.s001.doc]

**Table S1.** Summary characteristics of epiphytes and vines found in the surveyed forests. Data collected by the authors or reviewed from [60-61] and [36].

|  | **Type of growth** | **Vertical distribution**  (determines responses to edge proximity and patch shape) | **Main pollinators**  (determines responses to patch shape and tree size) | **Main dispersers**  (determines responses to patch size and isolation) |
| --- | --- | --- | --- | --- |
| *Luzuriaga polyphilla* | Secondary hemiepiphyte | 0-3 m | Insects | Birds + Marsupial |
| *Luzuriaga radicans* | Secondary hemiepiphyte | 0-3 m | Insects | Birds + Marsupial |
| *Campsidium valdivianum* | Vine | 4-8 m | Hummingbird | Wind |
| *Asteranthera ovate* | Secondary hemiepiphyte | 3-6 m | Hummingbird | Marsupial |
| *Sarmienta repens* | Holoepiphyte | 6-12 m | Hummingbird | Marsupial |
| *Mitraria coccinea* | Secondary hemiepiphyte | 3-6 m | Hummingbird | Marsupial |
| *Tristerix corymbosus* | Hemiparasitic mistletoe | 3-6 m | Hummingbird | Marsupial |
| *Fascicularia bicolor* | Holoepiphyte | 3-6 m | Insects | Birds |
