# Supplementary material for: Internal Habitat Quality Determines the Effects of Fragmentation on Austral Forest Climbing and Epiphytic Angiosperms
Source: PLoS One. 2012 Oct 31;7(10):e48743. doi: 10.1371/journal.pone.0048743 (PMC3485344; doi:10.1371/journal.pone.0048743)
Supplement: Table S2 — Values for some of the variables measured at the patch level for each of the 18 patches sampled. (DOC) [file pone.0048743.s002.doc]

**Table S2.** Values for some of the variables measured at the patch level for each of the 18 patches sampled.

| Fragment | Area (ha) | P/A Ratio | Circle | Distance to the nearest neighbor (m) | Proximity (100-m-buffer) | Proximity (500-m-buffer) | Density of main pollinator (ind/ha) | Density of main dispersers (ind/ha) | Mean DBH | Mean number of trees / 10 m |
| --- | --- | --- | --- | --- | --- | --- | --- | --- | --- | --- |
| Anibal | 2.03 | 0.036 | 0.40 | 66.3 | 7.95 | 24.42 | 64.04 | 13.65 | 14.58 | 4.40 |
| Arizmendi | 18.36 | 0.014 | 0.45 | 129.39 | 0.00 | 1525.02 | 11.78 | 26.86 | 17.77 | 8.55 |
| Canelo | 136.78 | 0.009 | 0.55 | 0 | 0.00 | 1253.61 | 72.97 | 9.29 | 10.87 | 7.60 |
| Duran | 519.68 | 0.007 | 0.64 | 0 | 28543.12 | 28581.97 | 32.17 | 9.11 | 12.51 | 6.35 |
| Gallardo | 51.17 | 0.011 | 0.62 | 40.98 | 1091.94 | 1103.67 | 52.79 | 13.11 | 16.48 | 5.05 |
| Hella | 10.80 | 0.021 | 0.70 | 26.13 | 3018.84 | 3021.35 | 96.17 | 21.47 | 17.13 | 7.18 |
| Juvenal | 3.49 | 0.031 | 0.53 | 65.4 | 4.85 | 14.47 | 57.76 | 19.91 | 14.49 | 6.72 |
| Koch | 2465.99 | 0.006 | 0.67 | 0 | 6061.14 | 6216.84 | 85.13 | 23.19 | 17.25 | 7.08 |
| Konpatski | 5.20 | 0.024 | 0.54 | 599.24 | 0.00 | 0.00 | 25.2 | 21.64 | 20.54 | 5.52 |
| Konpatski II | 30.54 | 0.011 | 0.54 | 28.09 | 13.16 | 17.71 | 17.37 | 11.12 | 13.92 | 6.88 |
| Linebrick | 14.05 | 0.017 | 0.56 | 40.05 | 156.92 | 165.10 | 47.49 | 12.34 | 16.20 | 5.05 |
| Minert | 25.59 | 0.013 | 0.48 | 124.08 | 0.00 | 43.75 | 28.49 | 9.13 | 14.14 | 6.20 |
| Ojeda | 9.58 | 0.020 | 0.68 | 114.34 | 0.00 | 19.92 | 73.15 | 7.24 | 15.43 | 6.25 |
| Ojeda U | 3.98 | 0.027 | 0.55 | 114.34 | 0.00 | 10.50 | 46.11 | 8.93 | 14.42 | 3.63 |
| Ostracodo | 108.84 | 0.008 | 0.58 | 167.74 | 0.00 | 464.00 | 26.14 | 12.17 | 13.67 | 7.62 |
| Rapoport | 175.61 | 0.011 | 0.70 | 0 | 27.01 | 148.52 | 81.95 | 6.41 | 13.52 | 6.80 |
| Ulloa-Delgado | 24.37 | 0.019 | 0.69 | 121.94 | 0.00 | 427.09 | 22.69 | 13.07 | 14.61 | 10.60 |
| Zuñiga | 5.52 | 0.023 | 0.51 | 56.77 | 13.50 | 1501.20 | 69.77 | 20.60 | 14.87 | 8.95 |
