# Supplementary material for: Internal Habitat Quality Determines the Effects of Fragmentation on Austral Forest Climbing and Epiphytic Angiosperms
Source: PLoS One. 2012 Oct 31;7(10):e48743. doi: 10.1371/journal.pone.0048743 (PMC3485344; doi:10.1371/journal.pone.0048743)
Supplement: Table S3 — Spatial autocorrelation analyses. Correlograms showing Morańs I statistic as a function of the distance between trees inside patches (distances ranging from 0 to 100 m) and between total patches (distances ranging from 0 to 20 km). a) L. radicans b) L. polyphylla c) M. coccinea d) S. repens e) C. valdivianum f) A. ovata. (DOC) [file pone.0048743.s003.doc]

**Table S3.** Spatial autocorrelation analyses. Correlograms showing Moran´s I statistic as a function of the distance between trees inside patches (distances ranging from 0 to 100 m) and between total patches (distances ranging from 0 to 20 km). a) *L. radicans* b) *L. polyphilla* c) *M. coccinea* d) *S. repens* e) *C. valdivianum* f) *A. ovata*.

| **a) Raw data** | **GLMM Model Residuals** |
| --- | --- |
|  |  |
|  |  |

| **b) Raw data** | **GLMM Model Residuals** |
| --- | --- |
|  |  |
|  |  |
| **c) Raw data** | **GLMM Model Residuals** |
|  |  |
|  |  |

| **d) Raw data** | **GLMM Model Residuals** |
| --- | --- |
|  |  |
|  |  |
| **e) Raw data** | **GLMM Model Residuals** |
|  |  |
|  |  |

| **f) Raw data** | **GLMM Model Residuals** |
| --- | --- |
|  |  |
|  |  |
